# Possible Shifts in the Genetic Diversity of Red-crowned Cranes (*Grus japonensis*) in Hokkaido, Japan: Indications of Continental Gene Flow

**DOI:** 10.3390/ani14111633

**Published:** 2024-05-30

**Authors:** Wenjing Dong, Kai Tomita, Akira Sawada, Makoto Hasebe, Masako Inoue, Kunikazu Momose, Tatsuro Nakamura, Hiroki Teraoka

**Affiliations:** 1 School of Veterinary Medicine, Rakuno Gakuen University, Ebetsu 069-8501, Japan; dongwenjing_2015@163.com (W.D.); s21861114@g.rakuno.ac.jp (K.T.); t-naka@rakuno.ac.jp (T.N.); 2 Faculty of Human Sciences, Waseda University, Tokorozawa 359-1192, Japan; akira.sawada.1312@gmail.com; 3 NPO Sarobetsu Eco Network, Toyotomi 098-4100, Japan; hasebe@sarobetsu.or.jp; 4 NPO Red-crowned Crane Conservancy, Kushiro 085-0036, Japan; masako@seagreen.ocn.ne.jp (M.I.); momosekunikazu@gmail.com (K.M.)

**Keywords:** genetic diversity, *Grus japonensis*, Hokkaido, eastern Eurasia, MHC, Red-crowned crane

## Abstract

**Simple Summary:**

The red-crowned cranes consist of two populations: continental (Far East Eurasia) and island (Hokkaido, Japan) cranes. It was once thought that these two populations lived independently. Having recovered from near extinction more than a century ago, the island population in Hokkaido, Japan, now exhibits very low genetic diversity, raising concerns about potentially devastating effects from infectious diseases. In 2018, a possible mating between a continental male and an island female was observed in northern Hokkaido. This study investigates their offspring or their blood relatives by examining the major histocompatibility complex (MHC) in cranes from northern and southeastern Hokkaido between 2008 and 2022. We identified 58 MHC types based on nucleotide sequences. MHC types from the possible continental male were predominantly found in cranes from northern Hokkaido. Genetic analysis also suggested clear population differentiation between northern and southeastern Hokkaido. The results suggest that genetic traits from the continental population have been integrated into the Hokkaido cranes, particularly in the northern population. This genetic exchange may improve the disease resistance and environmental adaptability of the Hokkaido crane population, providing valuable insights for conservation efforts.

**Abstract:**

Red-crowned cranes (*Grus japonensis*) consist of two distinct groups: the continental population and the island population. The island population, localized in Hokkaido, Japan, exhibits very low genetic diversity due to its rapid recovery from the brink of extinction. Our previous research in 2018 highlighted a possible mating between a male from the continental population, with the Gj5 haplotype, and a female from the island population, with the Gj2 haplotype, at Hitominuma Sawmp shore in northern Hokkaido. The present study attempted to unravel the distribution of their offspring by examining the major histocompatibility complex (MHC) of this mixed breeding pair compared with samples collected from cranes in northern and southeastern Hokkaido between 2008 and 2022. The analysis identified 55 MHC types, including 10 known types in a dataset of 89 crane samples, based on amino acid sequences. A total of 58 MHC types were recognized, based on nucleotide sequences, as there were many cases in which the same amino acid sequence had different nucleotide sequences. The five DNA types of MHC in the Hitominuma Swamp male were predominantly identified in eight cranes from northern Hokkaido and one chick from southeastern Hokkaido. In addition, population genetic analysis, based on insertion/deletion (InDel) polymorphisms, indicates distinct population differentiation between the northern and southeastern regions of Hokkaido. These results suggest that genetic contributions from the continental red-crowned crane population have already been integrated into the Hokkaido populations, with a more pronounced influence in northern Hokkaido.

## 1. Introduction

**Figure 1:**
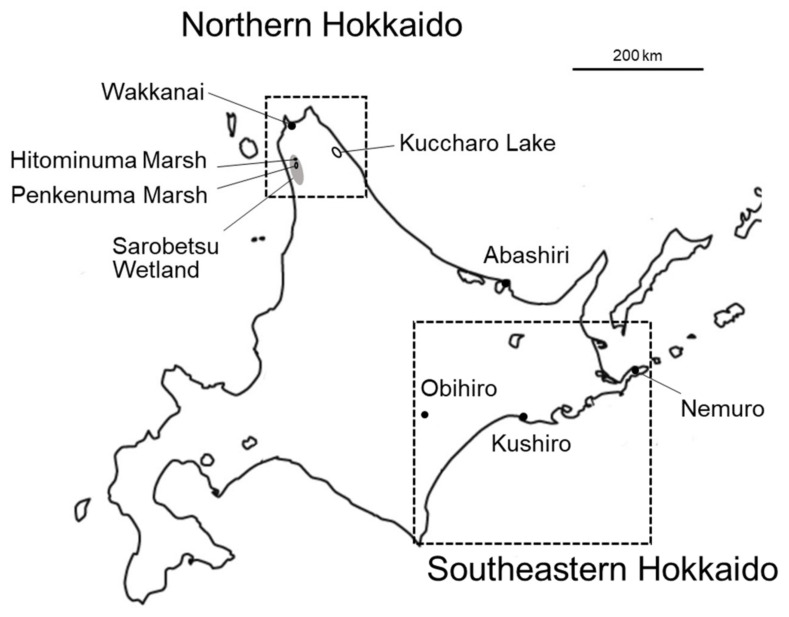
Simple map of Hokkaido, Japan. Dotted squares indicate northern Hokkaido and southeastern Hokkaido, respectively.

The red-crowned crane (*Grus japonensis*) is a large wetland bird species classified as “vulnerable” by the International Union for Conservation of Nature (IUCN), with an estimated population of about 4000 individuals [1]. There are two distinct populations of red-crowned cranes: the migratory continental population and the resident island population (Hokkaido population). The continental population breeds in the vast wetlands of the Amur River basin and migrates seasonally to wintering grounds, including the Demilitarized Zone (DMZ) between North and South Korea and parts of southeastern China. Conversely, cranes in the Hokkaido population are non-migratory and reside primarily in the southeastern areas of Hokkaido, Japan (Figure 1) [2].

Historically, the island population of red-crowned cranes in Hokkaido migrated to Honshu Island for wintering [3]. However, during the confusion of the early Meiji period (1868–1912), overhunting and the reduction of marshland habitats are thought to have forced these birds to settle in southeastern Hokkaido. By the end of the Meiji period, it was feared that the red-crowned crane had become extinct in Hokkaido [4]. However, the population has made a remarkable recovery, increasing from about 40 survivors to the current count of about 1800 individuals, largely due to corn provided by humans [2,5]. This rapid population growth from a small pool of survivors has resulted in a population with low genetic diversity, which poses a major concern for conservation [6,7,8,9].

In northern Hokkaido, red-crowned cranes were present until about 1860, but there have been no recorded sightings since then [10]. However, since the discovery of a pair of red-crowned cranes in the Sarobetsu Wetlands in 2002, cranes have been identified in Wakkanai and Lake Kutcharo (Figure 1). Aircraft surveys during 2003–2015 revealed that nesting adults and chicks have frequently been observed in North Hokkaido since 2004, a total of 13 cranes were observed around Penkenuma Swamp, and a total of 9 cranes were seen around Lake Kuccharo during the period 2004–2015. A total of 13 chicks were identified in the Penkenuma Swamp area, and 9 chicks were observed in the Lake Kuccharo area during the period 2004–2015. The chick population in northern Hokkaido is currently increasing [10].

To date, 18 haplotypes have been identified within the D-loop region of the red-crowned crane mitochondrial DNA, designated as Gj1 to Gj18, including one potential duplicate sequence [9]. Most of these have been found in continental individuals or in dozens of red-crowned cranes of continental origin in captivity (Gj3–Gj12, Gj14–18), and only two types, Gj1 and Gj2, have been identified in Hokkaido individuals, despite studies of several hundred birds [6,7,8,9]. While Gj13, the third Hokkaido type, was reported by Akiyama et al. [8], it was detected in only two birds, including a stuffed crane, and it is already considered extinct. An examination of microsatellite [11] and InDel (insertion/deletion) types [12] also supported the very low genetic diversity of the Hokkaido population. It is thought that this may be the result of a bottleneck effect stemming from an extended period of near extinction from the late Meiji period to the Taisho period (circa 1912) [6,7].

Based on haplotype analysis, it has been reported that three continental red-crowned cranes arrived in Hokkaido for brief periods in the 2000s [13]. In July 2018, an abandoned red-crowned crane nest, containing several feathers and an unfertilized egg, was found on the south bank of Hitominuma Swamp, located in the Sarobetsu Wetlands in northern Hokkaido (Figure 1). An analysis of feathers collected from the nest indicated that a male with a continental haplotype (Gj5) had mated with a female with a Hokkaido haplotype (Gj2). This discovery provided the first evidence that individuals from the continental population may have reached Hokkaido and successfully interbred with the local Hokkaido population [12]. However, no information is currently available regarding the offspring of this pair.

The major histocompatibility complex (MHC) is crucial for the function of the immune system, especially for the activation of T cells [14]. The high polymorphism of MHC genes among individuals makes them a valuable tool for studies of genetic diversity [15,16]. Within the red-crowned crane population, Akiyama et al. and Xu et al. performed population analyses by sequencing the MHC class I exon 3 region [17,18]. Xu et al. identified 30 amino acid (AA) types of MHC specific for the continental population and 10 types belonging to the Hokkaido population [17].

MHC DNA types exhibit such remarkable diversity that they are used as markers to assess genetic variation [19,20]. In this study, we identified and classified the MHC types of red-crowned cranes in the northern and southwestern regions of Hokkaido, following a previous report that initially classified these types based on amino acids. Then, we compared the MHC DNA types of the Hitominuma Swamp pair in 2018 [9] with those of red-crowned cranes in northern and southeastern Hokkaido to investigate the possibility of the diffusion of continental-type genes into the island population.

## 2. Materials and Methods

### 2.1. Samples and DNA Extraction

The muscles of two adult cranes and blood samples of 56 chicks in southeastern Hokkaido were studied in our previous reports on the development of insertion/deletion markers of red-crowned cranes in southeastern Hokkaido [12]. Chick blood samples were collected in June and July of 2008–2021 during banding, with permission from the Japanese Ministry of the Environment (MOEJ: Tokyo, Japan) (MOEJI permission No. 1704261, 1704281, 1806126, 1806141, 1806151, 1906191). Breast muscles were collected from two adult cranes found dead in 2021 and were kept in a freezer in Kushiro Zoo, Kushiro, Hokkaido (Table S1). The sex and haplotype of eight feathers collected on the shores of Lake Kuccharo in 2010 were previously analyzed [7]. In this study, 21 feathers (all from 2021, except one collected in 2022) were newly collected in the Sarobetsu Wetlands in northern Hokkaido (Figure 1). The origin of these feathers was checked by the InDel method, along with the sex and haplotype (see below, Table S2). DNA was extracted from several pieces of the follicles or shafts of feathers (usually about 25 mg) using DNeasy Blood & Tissue Kits (Qiagen, Venlo, The Netherlands) [9]. The extracted DNA samples were stored at −20 °C until use. DNA samples previously extracted from eight feathers collected from the shore of Lake Kuccharo [7] and two feathers collected from the Hitominuma pair in 2018 [9] were also used. Sexing was performed by PCR-based methods using these DNA samples with two sets of primers, as described in our previous paper [9].

### 2.2. Genotyping of Major Histocompatibility Complex

We used high throughput sequencing of amplicons in our evaluation. To detect major histocompatibility complex (MHC) type, the same primer set flanking, MHC Class I Exon 3, as that used in a previous report [17] was employed here (MHC I-E3-F: 5′-TCAGCCCCRTCTCCCTGGTC-3′, MHC I-E3-R: 5′-GTAGAAGCCGTAAGCGCGGCA-3′). The first-round PCR was carried out with a DNA sample prepared as described in the previous section using Tks Gflex DNA Polymerase (Takara Bio, Kusatsu, Japan). We used a stepdown PCR protocol consisting of a stepdown procedure (94 °C for 60 s as initial denaturation, 3 cycles of denaturation at 98 °C for 10 s, annealing at 65 °C for 15 s, extension at 68 °C for 30 s; 3 cycles of denaturation at 98 °C for 10 s, annealing at 59 °C for 15 s, and extension at 68 °C for 30 s; 30 cycles of denaturation at 98 °C for 10 s, annealing at 53 °C for 15 s, and extension at 68 °C for 120 s).

For addition of index sequences and adapter sequences to the first-round PCR products for identification of individual crane and high-throughput sequencing, respectively, the second-round PCR was carried out with the first-round PCR products using conjugate primer sets (MHCE3-F-A: TCGTCGGCAGCGTCAGATGTGTATAAGAGACAG [i7 Index Adapters] TCAGCCCCRTCTCCCTGGTC, MHC I-E3-R-A: GTCTCGTGGGCTCGGAGATGTGTATAAGAGACAG [i5 Index Adapters] GTAGAAGCCGTAAGCGCGGCA), according to 2-Step PCR Amplicon Library Preparation (Preparing Dual Index Amplicons Library for the Illumina MiSeq System) (Illumina, San Diego, CA, USA). Seven nucleotide index sequences (Index 1 Adapters, including N701, N702, N703, N704, N705, N706, N707, N710, N716, N718, N720, N723 (Index 1 Adapters), and S513, S515, S516, S517, S518, S520, S521, S522 (Index 2 Adapters), were supplied by Illumina (Illumina Adapter Sequences). The stepdown PCR protocol for the second-round PCR consists of a stepdown procedure (94 °C for 60 s as initial denaturation, 5 cycles of denaturation at 98 °C for 10 s, annealing at 55 °C for 15 s, extension at 68 °C for 30 s; 30 cycles of denaturation at 98 °C for 10 s, annealing at 59 °C for 15 s, and extension at 68 °C for 30 s; 30 cycles of denaturation at 98 °C for 10 s, annealing and extension at 68 °C for 45 s, and extension at 68 °C for 120 s). Aliquots of the PCR products were electrophoresed in 1% agarose gel, and ethidium bromide-stained bands were observed using a gel documentation system (WSE-5400: ATTO, Tokyo, Japan). Then, the remaining PCR products (amplicons) were purified with a Fast Gene TM Gel/PCR Extraction kit (NIPPON Genetics, Tokyo, Japan), and all amplicons were mixed for high-throughput amplicon sequencing (HTAS). After purification with AMpure XP (Beckman Coulter Life Sciences, Brea, CA, USA) and validation and quantification of the prepared samples with TapeStation (Agilent Technologies, Santa Clara, CA, USA), the mixed amplicons were sequenced with Illumina MiSeq PE (100 thousand reads/sample). All of the procedures were carried out according to the manufacturers’ instructions, unless otherwise noted.

Raw reads were trimmed using two programs, Cutadapt (https://cutadapt.readthedocs.io/en/stable/) (accessed on 1 March 2023) and Trimmomatic (https://github.com/usadellab/Trimmomatic) (accessed on 1 March 2023). Since sequencing was carried out with forward (Read1) and reverse (Read2) primers, respectively, the same sequences contained in the data were counted for the read pairs that were linked by Read1 and Read2 with fastq-join (https://expressionanalysis.github.io/ea-utils/) (accessed on 1 March 2023) to calculate the read counts of each sample. A read count with less than 0.1% of total read counts, or 10, was discarded to eliminate false positives due to index hopping [21].

The resulting reads were processed with AmplisSAT [22] to detect MHC variants. We firstly applied AmpliMERGE to the pair-end reads data, and the output files were passed to AmpliCHECK to summarize the reads and conduct trial genotyping. Since most of the putative variants yielded 450 bp, and sequences due to errors can hamper subsequent genotyping analysis, we filtered out sequences shorter than 434 bp and longer than 466 bp (sequences with 450 ± 15 bp were retained). Then, AmpliSAS was applied to the read file after the filtering. The parameter “maximum number of alleles per amplicon” was set to 15, based on the recommendations of Akiyama et al. [18] and Xu et al. [17], who detected a maximum of 11 variants per individual. The parameter “minimum amplicon depth” was set to 20, based on the results of AmpliCHECK, in which variants with a depth less than 20 were detected in only one sample, likely due to artifacts. Here, MHC variants detected in this study cannot be assigned to specific loci in their genome. Therefore, although classifying them as alleles is not appropriate in the strict sense, we call them such for convenience.

### 2.3. Genotyping of Mitochondrial Haplotype

The mitochondrial haplotype (control region 2, CR2) was conveniently determined using the amplification refractory mutation system (ARMS) PCR assay with the same DNA extracts from the blood samples and the muscle tissues as templates [7]. The ARMS method, capable of detecting Gj1, Gj2, Gj13, and other haplotypes, is nearly sufficient, as all cranes in Hokkaido can be classified into these three haplotypes, except for very rare individuals, possibly of continental origin [7]. Haplotypes of some samples were confirmed by direct Sanger sequencing.

### 2.4. Genotyping InDels

Using our 11 designed InDel (insertion/deletion) primer sets and DNA extracts from feather, blood, and a muscle as templates, PCR reactions were carried out with GoTaq Green Master Mix (Promege, Fitchburg, WI, USA), as previously described in Ref. [12] (initial activation of heating at 95 °C for 120 s, 40 cycles of denaturation at 95 °C for 30 s, annealing at 60 °C for 30 s, extension at 72 °C for 30 s). Electrophoresis with 3% agarose gel was carried out, and patterns of positive bands were visualized with application of 10 µg/mL ethidium bromide on the gel plate. PCR products were extracted from these positive bands with the FastGene Gel/PCR Extraction Kit (Nippon Genetics, Tokyo, Japan) and used for direct Sanger sequencing for confirmation of the specificity.

### 2.5. Statistical Analyses

MHC-type data and InDel-type data for 31 non-kin individuals for southeastern Hokkaido and 20 feathers for northern Hokkaido were used for basic population genetics. Although partially incomplete, individuals from feather samples were identified by InDel type, sex, and haplotype (see Section 3). To describe the basic population genetics of the cranes, observed heterozygosity (Ho), expected heterozygosity (He), and polymorphic information content (PIC) were calculated by Cervus [23]. Allelic diversity (AR) and F-statistics (Fst: measure of population differentiation, and F_IS_: measure of inbreeding) were estimated by SPAGeDi [24], based on the estimators of Weir and Cockerham [25]. Whether the F-statistics deviated from zero was tested using a permutation test implemented in SPAGeDi.

## 3. Results

### 3.1. Analysis of Insertion/Deletion in the Northern Hokkaido Population

The blood of chicks and breast muscles of adult red-crowned cranes collected in southeastern Hokkaido and feathers picked up on the shore of Lake Kuccharo in northern Hokkaido examined in this study have been analyzed in our previous studies [7]. In addition to these feathers, we reexamined the sex, haplotype, and insertion/deletion (InDel) type of feathers collected in the Sarobetsu Wetlands in 2021–2022 (Table S2). Possibly due to the low quantity of intact DNA that was extracted from some feathers, the frequencies of band detection for some primer sets were lower when compared to DNA extracts from blood, as reported by Kawasaki et al. [12]. Feather samples were used for the population genetic analysis for the northern population, excluding possible relatives by banding record or MHC type, as described below. In addition, the feathers of a continental-type Gj5 male in Hitominuma Swamp and Penkenuma Swamp 5, and the feathers of Gj2 female in Hitominuma Swamp and Penkenuma Swamp 13 recovered in 2018 were considered to be from the same individual, as their sex, haplotype, and InDel type were all matched (Table S2). Because of deficient information for some feather samples, the combinations of Penkenuma Swamp 10 and 11; Penkenuma 12, 14, and 17; Lake Kuccharo 1 and Penkenuma Swamp 6; Penkenuma Swamp 7 and Kabutonuma Swamp 2 were not confirmed as separate individuals by InDel type, sex, or haplotype.

Finally, a basic population analysis was carried out with 20 northern Hokkaido red-crowned cranes (samples from a Hitominuma Swamp Gj5 male, Gj2 female, No. 449, Kabutonuma Swamp 1–6, and Penkenuma Swamp 2, 4, 6–12, 14, 15, 17), excluding individuals that are possibly related (Table S2). Thus, several samples may have originated from the same individual, as mentioned above. The observed heterozygosity (Ho) [26], an indicator of genetic diversity, was 0.381 in the north Hokkaido population (Table 1). The inbreeding coefficient (F_IS_), which represents the degree of inbreeding, was −0.412, showing a significant deviation from zero (*p* = 0.0000).

Compared with the 31 southeastern individuals, excluding known blood relatives [12], the pairwise fixation index (Fst) in groups of northern and southeastern Hokkaido, based on InDel type, was 0.214387, which was also significantly different (*p* = 0.0000) (Table 2).

### 3.2. MHC Types Detected in Hokkaido Population

While MHC sequences of 150 bp or 274 bp were reported previously [17,18], our study using the new high-throughput sequencing (HTS) system was able to achieve sequence reads up to 420 bp (140 AA).

A total of 33 MHC types (amino acid sequences) were detected in a total of 89 samples from both northern and southeastern Hokkaido (Table S3), including blood relatives. A total of 10 of the 46 MHC (AA) types reported by Xu et al. [17] were confirmed in this study. These were all continent-specific (Grja-UA*27, 28, 33, 38, 44) or common to the continent and Japan (Grja-UA*47, 50, 51, 54, 55). On the other hand, 16 MHC types identified by Akiyama et al. in the southeastern Hokkaido population were not confirmed in this study [18]. We named the MHC examples for novel types, according to the suggestions of Xu et al. [17]. When the AA type of the MHC type was identical, but the nucleotide sequence was different, we added 1–5 to the AA type, in some cases. As shown in Table S3, we identified 23 novel MHC types from Grja-UA*57–79, with different sequence types (1 and 2) for Grja-UA*58–63. The majority of sequences up to the 98th AA matched the amino acid sequence of Grja-UA*27 (Table S3).

### 3.3. Distribution of MHC Types in Northern and Southeastern Hokkaido Populations

The percentage of detected MHC types (AA sequences) is shown for individuals from northern Hokkaido (blue) and southeastern Hokkaido (red) (Figure S1). Overall, Grja-UA*47a was the most abundant MHC type, followed by Grja-UA*28a, 50a, and 51a in Hokkaido. In particular, Grja-UA*47a was detected in more than 80% of the individuals in each population. Conversely, Grja-UA*71–79 was detected in only one crane (1.1%) of the total population. While Grja-UA*27, 44a, 67–70, 72–74, and 79 were found only in northern Hokkaido population, Grja-UA*28b, 47b, 61a, 63a, 64, 71, and 75–78 were found only in the southeastern Hokkaido population (Figure S1). Thus, there were substantial differences in the frequency of observed MHC types, as well as discernible differences between northern and southeastern populations. The Fst in two groups, based on the AA MHC type, was 0.012881 (*p* = 0.0000) (Table 2).

**Figure 2:**
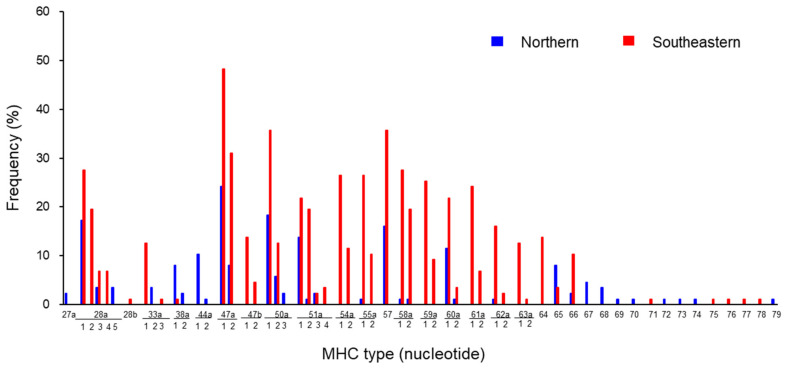
Frequencies of MHC class I exon 3 types based on the nucleotide sequence in the red-crowned crane in Hokkaido. Distributions and frequencies of each type in the northern (29 cranes, blue) and southeastern (58 cranes, red) population (total: 87 cranes). Several feather samples from the same individual may be included. For example, “27” means Grja-UA*27. To distinguish DNA MHC types with the same amino acid sequence but a different nucleotide sequence, “a1–a5” is added in the bottom row below 28a for UA*28a.

Since the main purpose of this study was not to study the functional aspect of MHC but to examine kinship, the detection frequency of the MHC sequence type was also expressed by the nucleotide sequence, as shown in Figure 2. Of the 32 feather samples from northern Hokkaido (Table S1), 29 were used to create a graph. The exceptions were the Hitominuma Swamp Gj5 male, whose MHC type was barely detected, and the Penkenuma Swamp 13, which appeared to originate from the Hitominuma Swamp Gj2 female. Therefore, as mentioned above, several feather samples from the same individual may be included. A total of 58 samples of all muscle and blood samples examined for MHC were included for Figure 2. The overall trend was similar to that of the amino acid sequence (Figure 1). The detection frequencies of the DNA MHC types in the red-crowned crane population varied widely, ranging from 50% (Grja-UA*47a1, 50a1, 57) to only 1.1% (Grja-UA*28b, 33a3, 44a2, 63a2, 71–79) in both southeastern and northern Hokkaido. Additionally, there was a marked bias in the detection frequency of each DNA MHC type between the populations of northern and southeastern Hokkaido. Particularly, Grja-UA*27, 28a5, 33a2, 38a2, 44a1, 44a2, 50a3, 67–70, 72–74, and 79 were detected only in the northern Hokkaido population.

### 3.4. Comparison of MHC Types in Parents and Children

Based on the results of a banding study carried out by the NPO Crane Conservation Research Group (CCR) over many years, we were able to determine the family relationships of the father (stock number R123 in the freezer), mother (R131), and their twins (banding numbers 244 and 245), and we compared the DNA MHC types of these families [12] (Table S4). The DNA MHC types of these individuals were confirmed: the father had 11 (blue), the mother had 8 (red), and the offspring had 10 and 7 MHC types, respectively. All DNA MHC types of the offspring were detected in either the father or the mother, except for Grja-UA*28a3 (highlighted in yellow), which may be due to a genotyping error. This supports the Mendelian inheritance of DNA MHC types in the red-crowned crane [27]. The percentages of these DNA MHC types were not high, with Grja-UA*54a2, 55a2, 59a2, 64, and 66 observed at 9.7–12.9% in 89 cranes in the Hokkaido population, suggesting that the concordance of these MHC types may not be coincidental.

### 3.5. MHC Types of Hitominuma Swamp Cranes in Northern and Southeastern Hokkaido Populations

In 2018, we searched for individual cranes (samples) with MHC types from the Gj5 male crane and the Gj2 female crane in a Hitominuma Swamp pair (Table 3). However, only one MHC type was confirmed in the feathers of the male Hitominuma Gj5 crane, most likely due to the limited amount of intact genomic DNA in the feather sample found in the field, which was subjected to environmental conditions of the wild. We adopted a feather of Penkenuma Swamp 5 that showed the same InDel type, sex, and haplotype as those of the Hitominuma Swamp male crane. Five DNA MHC types of Grja-UA*28a5, 38a1, 38a2, 67, and 68 were found in the male cranes of Hitominuma Swamp (Table 3). The frequencies of these five types were generally low, with the highest frequency being 8.6% for Grja-UA*38a1, and the rest being 4.3% or less. Eight cranes in northern Hokkaido (Lake Kuccharo 1, 4, and 6; Penkenuma Swamp 9; Kabutonuma Swamp 3, 5, 6, and 429 (Toyotomi)) possessed at least one of the same MHC types (blue) as the Gj5 male crane in Hitominuma Swamp. In southeastern Hokkaido, Grja-UA*38a1, found in the Gj5 Hitominuma male crane, was also confirmed in one crane (315) caught for banding in Hamanaka Town in July 2017, as the only crane in southeastern Hokkaido. These nine cranes also shared at least two of the same MHC types (red) as the Gj2 female in Htominuma Swamp, although some of these DNA MHC types are not rare in the Hokkaido population (Table 3).

## 4. Discussion

We have previously reported that PCR using 11 different InDel primer sets can identify individual cranes with high accuracy [12]. In addition, when sex and haplotype information are added, it becomes possible to determine, with very high accuracy, whether feather samples are from the same individual. However, due to the limited amount of DNA that could be extracted from the feathers and the severe environmental conditions in the field, some InDel types and sexes could not be determined, especially in the Penkenuma Swamp samples. As a result, we cannot rule out the possibility that some of the feather pairs may have come from the same individual. However, since the samples from Lake Kuccharo were collected in 2010, and those from Kabutonuma Swamp and Penkenuma Swamp were collected in 2021–2022, it is unlikely that the Lake Kuccharo 1 and Penkenuma Swamp 6 samples are from the same individual. In addition, since the feather collection sites of Kabutonuma Swamp and Penkenuma Swamp are about 20 km apart, it is also unlikely that the Penkenuma Swamp 7 and Kabutonuma Swamp 2 samples are from the same crane. A red-crowned crane family was observed on the shores of Kabutonuma Swamp in the summer of 2022 [28]. We collected all feathers around the peninsular mouth of Penkenuma Swamp, although the locations are scattered. Three pairs of red-crowned cranes were observed in Penkenuma Swamp at that time [28]. Therefore, we must consider the possibility that the feather samples collected in Penkenuma Swamp (combinations of Penkenuma Swamp 10 and 11, and Penkenuma 12, 14, and 17) may have come from the same individual. We admit that this could affect the population analysis and the frequency distribution of MHC types, to some extent.

Xu et al. [17] identified three different DNA types of MHC: those specific to the continents, those specific to Hokkaido, and those that are common to both regions. In this study, among the 35 MHC types identified in 89 Hokkaido cranes (northern and southeastern Hokkaido), three types (Grja-UA*27, 33, and 38) were considered to be continent-specific. None of the six MHC types that were thought to be unique to Hokkaido were found. Several cases were found in which the nucleotide sequences were different, even though the deduced amino acids were perfectly matched; Akiyama et al. [18] and Xu et al. [17] identified sequences of up to 91 AA, while our study extended this to determine sequences of up to 140 AA. Consequently, we observed cases in which sequences matched previous findings up to the 91st amino acid, but showed variations thereafter. The total number of DNA MHC types detected in this study was 58.

We reported that a male feather found in 2018 at a nest on the south bank of Hitominuma Swamp in the Sarobetsu Wetlands of northern Hokkaido was of continental origin, based on the haplotype (Gj5) [9]. Only one MHC type could be identified from the DNA extracted from this feather, but among the 14 feathers collected around Penkenuma Swamp on 8 September 2021, a Gj5 male was found whose InDel type perfectly matched that of a Gj5 male in Hitominuma Swamp. Therefore, this MHC type was considered to be that of the Gj5 male in Hitominuma Swamp. We reported a 99.9% probability of individual identification, based on a comparison of the 11 InDel types used in this study [12]. It is highly unlikely that another Gj5 male crane lived in close proximity to Hitominuma Swamp. Five MHC types detected in a Gj5 male in the Hitominuma Swamp were relatively rare, accounting for 2.2–4.3% of the Hokkaido population studied in this research, except for Grja-UA*38a1 (8.6%). Among them, Grja-UA*38a has already been identified as a continental type by Xu et al. [17]. These five MHC types were identified in six birds from the northern Hokkaido population and one bird from Hamanaka Town in southeastern Hokkaido, respectively. All the feathers, except the one from Lake Kuccharo, were collected after 2021. However, without knowing the age of the bird that left the feather, we cannot exclude the possibility that this bird could be the progenitor of Hitominuma Gj5 male, a crane that reached adulthood by 2018.

Three feathers from the shores of Lake Kuccharo, collected in 2009, had at least one of the same MHC types as the Gj5 male in Hitominuma Swamp. All feathers from Lake Kuccharo were identified as Gj2 [7]. Despite a thorough examination of hundreds of cranes by the Hokkaido University group and our team, the Gj5 has not been identified in any female in the Hokkaido population [9]. According to Masatomi et al. [10], the first nesting was observed in the Sarobetsu Wetland in 2004. Unfortunately, no samples are currently available, but we have confirmed the presence of a female Gj2 individual from breast feathers collected in the Sarobetsu Wetland [7].

On the other hand, there are records of successful breeding in red-crowned cranes up to the age of 29 years in males and 27 years in females [29]. Therefore, it is also possible that the family group observed at Lake Kuccharo in 2009 may be descendants of the breeding pair at Hitominuma Swamp in 2018. Since MHC follows Mendelian inheritance [27], which was confirmed for the red-crowned crane in this study, it is thought that the MHC type of the offspring is inherited from either parent. This is not easily determined because of the limitations of detecting all MHC types in individuals using HTS, which can vary depending on the condition of the feathers. The longer MHC sequences analyzed in this study may also account for the lower frequency of detection. In any case, it is important to note that of the nine individuals that shared MHC types with the Hitominuma Gj5 male, each also possessed three to six unique MHC types not found in the Hitominuma Swamp pair. One feather sample collected in Kabutonuma Swamp (Kabutonuma 3) matched all the MHC types of the Hitominuma Swamp pair, but only three MHC types were confirmed in total. In addition, other cranes from Kabutonuma Swamp (Kabutonuma 5) and No. 315 had three identical types of MHC and two types of MHC different from those of the Hitominuma Swamp pair. Therefore, we could not conclude the presence of offspring from the Hitominuma Swamp pair in this study. In any case, considering that the parent of the Hitominuma Gj5 male is unlikely to be a Hokkaido individual [9], and taking into account the limited number of MHC types identified in the Hitominuma Gj5 male in the southeastern Hokkaido population, it is suggested that genes from the continental population have already spread mainly to the northern regions of Hokkaido.

The only unique MHC type of the Hitominuma Gj5 male was found in a single chick (No. 315) banded in 2017 in Hamanaka Town, southeastern Hokkaido. Considering that chicks unable to fly have a limited range of movement, it is likely that they were born near where they were captured. If the Grja-UA*65 type was not originally part of the Hokkaido population, at least one of its parents must have possessed the continental MHC type. The crane with banding No. 426 was one of the first four birds (No. 426–429) banded in northern Hokkaido in 2021 and 2022 (Toyotomi Town) [28]. Based on the transmitter record, crane No. 426 migrated to Tsurui Village in southeastern Hokkaido after spending about a month on the shore of Lake Kuccharo [28]. After the first observation of a pair of red-crowned cranes in Sarobetsu Wetland in 2004 [7], there have been no records of red-crowned cranes in the area during winter thus far [10]. Based on this information, it is likely that the northern Hokkaido population overwinters near some feeding stations in southeastern Hokkaido. Thus, if cranes of continental origin are present in northern Hokkaido, they would naturally encounter the southeastern Hokkaido or Abashiri population (Figure 1) at several feeding grounds in southeastern Hokkaido while overwintering [2]. Therefore, it is possible that continental-type genes have already spread to southeastern Hokkaido.

On the other hand, the Gj2 of the Hitominuma female was very popular in the Hokkaido population, both in the northern and other parts of Hokkaido. We are not aware of any extensive haplotype studies in continental populations, but we have not found any reports of detection of the Gj2 type on the continent [9]. In addition, based on haplotype study using feathers, continental-type red-crowned cranes have appeared in Hokkaido and Honshu in the past [13]. However, it is noteworthy that all identified continental-type cranes were males [13]. This finding is consistent with the current study, which also documented a male continental-type crane in Hitominuma Swamp. Recently, we suggested again that the MHC type Gj2 belongs to the Hokkaido population [9], as is also the case for the suggestions of previous reports [6,7,8]. Indeed, Gj2 is the predominant haplotype within the Hokkaido population, accounting for over 90% of individuals [9]. Our results emphasize the predominance of the Gj2 haplotype, not only in southeastern Hokkaido and the Abashiri area, but also in the northern population of red-crowned cranes.

Using microsatellites as a genetic marker, Sugimoto et al. examined the degree of population differentiation in Tokachi, Kushiro, and Nemuro, indicating some degree of differentiation, with Tokachi and Nemuro showing a relatively low degree of differentiation [11]. Conversely, according to population analysis using the InDel typing [12], no significant differentiation was observed in Tokachi and Nemuro, although there is a noticeable trend. Therefore, the southeastern Hokkaido population (Kushiro, Nemuro, and Tokachi regions) was treated as a single population in the population analysis. The MHC type and InDel type were used separately for analysis. However, since the MHC variants cannot be assigned to specific loci and thus, we cannot obtain precise allele frequencies, the analysis using MHC types was not significantly reliable; therefore, it was used as a reference. On the other hand, there were concerns about the accuracy of the analysis for InDel typing, as a number of PCR bands were not detected. This may be due to the fact that, except for the blood of Nos. 429 and 492, all other samples in the northern Hokkaido consisted of feathers found on the ground. However, we obtained sufficient pairwise Fst values and corresponding p values from the G-test for genetic differentiation between groups of southeastern and northern Hokkaido based on InDel type. These results indicate that there are significant genetic differences between the populations from southeastern and northern Hokkaido. The inbreeding coefficient (Fis) reflects the degree of inbreeding in a population, and Fis values of 0 and 1 indicate free mating and complete inbreeding, respectively [26]. The southeastern Hokkaido population showed a positive but non-significant Fis value of 0.095 (*p* = 0.075) [12]. Although the result did not reach the threshold for statistical significance, it suggests a tendency toward inbreeding within this population. In contrast, the northern Hokkaido population showed a significant negative Fis value of −0.412 (*p* = 0.0000). This suggests a higher number of heterozygous individuals than would be expected from random mating alone. The low fecundity of small populations may result in negative Fis values when hybridization occurs with individuals from outside populations [30]. Since all haplotypes of the northern Hokkaido population are Gj2, it is thought that they originally diverged from the southeastern Hokkaido or Abashiri populations and settled in the Sarobetsu Wetland around 2002 [7,10]. Therefore, the results indicated the possibility that individuals from the mainland may have invaded and interbred with the northern Hokkaido population, which is derived from the southeastern Hokkaido or Abashiri population, with low genetic diversity and a tendency to inbreeding.

The fixed index (Fst) is a measure of the degree of genetic differentiation between populations due to inbreeding; it ranges from 0 to 1, with higher values indicating greater differentiation. An Fst of 0 indicates no difference between populations, while an Fst of 1 indicates complete differentiation, and an Fst of 0.15 is considered to show significant differentiation [26]. The Fst of the northern and southeastern Hokkaido populations was 0.214387 (*p* = 0), indicating a significant degree of differentiation, although the degree was not large. Considering the previous paragraph, this result is further suggested that the observed genetic differences between the two populations may be due to interbreeding with individuals from the continent.

Common cranes (*Grus grus*), which share part of their habitat with red-crowned cranes in the mainland, have been observed several times in southeastern Hokkaido [31]. Therefore, it is not surprising that red-crowned cranes flying from Honshu may fly directly to feeding grounds in southeastern Hokkaido for overwintering and then remain there [9].

## 5. Conclusions

In this study, we did not obtain conclusive evidence for the presence of offspring from a mating pair consisting of a continental-type male (Gj5) and a Hokkaido-type female (Gj2) discovered in 2018 in Hitominuma Swamp, northern Hokkaido [9]. However, we identified several red-crowned crane individuals, particularly in northern Hokkaido, that appear to be genetically related to the Hitominuma Swamp Gj5 male crane. One MHC type of the continental male (Gj5) was also detected in chicks in southeastern Hokkaido, suggesting that genes of continental origin may have spread to southeastern Hokkaido, to some extent. To date, all red-crowned cranes presumed to be of mainland origin have been identified as males. This observation may indicate sex dependency in the nesting site exploration behavior of red-crowned cranes, providing valuable insights for potential conservation strategies in the future. Therefore, further analysis of the MHC is needed to understand this pattern in more detail.

## Figures and Tables

**Table 1:** Some parameters of population genetics in the northern and southeastern population using InDel markers and MHC markers.

		N	AR	He	Ho	Fis	P
InDel	Total	51	1.90	0.3352	0.315	0.062	0.1868
	Northern	20	1.71	0.2737	0.381	−0.412	0.0000
	Southeastern	31	1.90	0.3088	0.282	0.090	0.1139
MHC	Total	51	34.09	0.9513	1.00	−0.044	0.0000
	Northern	20	33.00	0.9221	1.00	−0.075	0.0000
	Southeastern	31	30.55	0.9606	1.00	−0.041	0.0000

Non-kin cranes were selected from northern and southeastern populations. For northern population (Northern), however, several feather samples from the same individual may be included (see text). N: observed number of crane individuals, AR: allelic diversity, Ho: observed heterozygosity, He: expected heterozygosity, Fis: the inbreeding coefficient in F-statistics, P: corresponding *p* values in the permutation test.

**Table 2:** Pairwise Fst values and corresponding p values from the permutation test for genetic differentiation between groups of northern and southeastern Hokkaido, based on InDel type and MHC type.

	Fst	P
InDel	0.214387	0.0000
MHC	0.012881	0.0000

Fst: pairwise fixation index; P: corresponding *p* values in the permutation test; N = 51.

**Table 3:** Individual red-crowned crane with MHC type (nucleotide) detected in the Gj5 male in Hitominuma Swamp.

	Grja-UA*	28a1	28a3	28a5	33a2	38a1	38a2	44a1	44a2	47a1	47a2	50a1	50a2	50a3	51a1	57	59a1	60a1	60a2	65	67	68	69	74	76
Hitominuma male																									
Hitominuma female																									
	% northern	48.6	8.6	8.6		20	5.7					51.4	14.3					28.6	2.9	20	11.4	8.6			
	% southeastern	41.4	10	0		1.7	0					53.4	19					32.8	5.2	5.2	0	0			
	% of total	44.1	9.7	3.2		8.6	2.2					52.7	17.2					31.2	4.3	10.8	4.3	3.2			
Lake Kuccharo 1																									
Lake Kuccharo 4																									
Lake Kuccharo 6																									
Penkenuma 9																									
Kabutonuma 3																									
Kabutonuma 5																									
Kabutonuma 6																									
429																									
315																									

Blue boxes indicate that the same MHC type (nucleotide) was detected as that in the Hitominuma swamp male (Gj5) (Hitominuma male). Red boxes indicate the same MHC type as that in the Hitominuma swamp female (Gj2) (Hitominuma female). “% of total cranes” represents the detection rate among 89 cranes examined in this study. Purple boxes indicate the same MHC type as that of the Hitominuma pair in 2018, and yellow boxes indicate no MHC type matching this pair. MHC types of this pair were not detected in the other cranes. For example, “*28a1” in the top row means Grja-UA*28a1. Penkenuma and Kabutonuma indicate Penkenuma Swamp and Kabutonuma Swamp, respectively. The MHC types of Penkenuma Swamp 5 were used instead of that of the Hitominuma Gj5 male, since their origin was thought to be the same (see text).

## Data Availability

The datasets generated and/or analyzed during the current study are available from the corresponding author on reasonable request.

## Supplementary Materials

The following supporting information can be downloaded at: https://www.mdpi.com/article/10.3390/ani14111633/s1, Table S1. Muscle and blood samples collected in southeastern Hokkaido used in this study; Table S2. InDel type of feathers and bloods collected in northern Hokkaido between 2010 and 2021; Table S3. Amino acid sequences of MHC class I exon 3 molecules from red-crowned cranes in Hokkaido, Japan; Table S4. Comparison of nucleotide type of MHC in a family of red-crowned crane in southeastern Hokkaido; Figure S1. Frequencies of MHC class I exon 3 types based on AA sequence in red-crowned crane in Hokkaido.
