# Correction: Dong et al. Possible Shifts in the Genetic Diversity of Red-crowned Cranes (*Grus japonensis*) in Hokkaido, Japan: Indications of Continental Gene Flow. *Animals* 2024, *14*, 1633

**DOI:** 10.3390/ani14182639

**Published:** 2024-09-11

**Authors:** Wenjing Dong, Kai Tomita, Akira Sawada, Makoto Hasebe, Masako Inoue, Kunikazu Momose, Tatsuro Nakamura, Hiroki Teraoka

**Affiliations:** 1 School of Veterinary Medicine, Rakuno Gakuen University, Ebetsu 069-8501, Japan; dongwenjing_2015@163.com (W.D.); s21861114@g.rakuno.ac.jp (K.T.); t-naka@rakuno.ac.jp (T.N.); 2 Faculty of Human Sciences, Waseda University, Tokorozawa 359-1192, Japan; akira.sawada.1312@gmail.com; 3 NPO Sarobetsu Eco Network, Toyotomi 098-4100, Japan; hasebe@sarobetsu.or.jp; 4 NPO Red-crowned Crane Conservancy, Kushiro 085-0036, Japan; masako@seagreen.ocn.ne.jp (M.I.); momosekunikazu@gmail.com (K.M.)

## Error in Table

In the original publication [1], there was a mistake in Table 3 as published. The column for Hitominuma female 67 was incorrectly labeled as white, whereas it should have been red. The corrected Table 3 appears below. The authors state that the scientific conclusions are unaffected. This correction was approved by the Academic Editor. The original publication has also been updated.

## Figures and Tables

**Table 3:** Individual red-crowned crane with MHC type (nucleotide) detected in the Gj5 male in Hitominuma Swamp.

	Grja-UA*	28a1	28a3	28a5	33a2	38a1	38a2	44a1	44a2	47a1	47a2	50a1	50a2	50a3	51a1	57	59a1	60a1	60a2	65	67	68	69	74	76
Hitominuma male																									
Hitominuma female																									
	% northern	48.6	8.6	8.6		20	5.7					51.4	14.3					28.6	2.9	20	11.4	8.6			
	% southeastern	41.4	10	0		1.7	0					53.4	19					32.8	5.2	5.2	0	0			
	% of total	44.1	9.7	3.2		8.6	2.2					52.7	17.2					31.2	4.3	10.8	4.3	3.2			
Lake Kuccharo 1																									
Lake Kuccharo 4																									
Lake Kuccharo 6																									
Penkenuma 9																									
Kabutonuma 3																									
Kabutonuma 5																									
Kabutonuma 6																									
429																									
315																									

Blue boxes indicate that the same MHC type (nucleotide) was detected as that in the Hitominuma swamp male (Gj5) (Hitominuma male). Red boxes indicate the same MHC type as that in the Hitominuma swamp female (Gj2) (Hitominuma female). “% of total cranes” represents the detection rate among 89 cranes examined in this study. Purple boxes indicate the same MHC type as that of the Hitominuma pair in 2018, and yellow boxes indicate no MHC type matching this pair. MHC types of this pair were not detected in the other cranes. For example, “*28a1” in the top row means Grja-UA*28a1. Penkenuma and Kabutonuma indicate Penkenuma Swamp and Kabutonuma Swamp, respectively. The MHC types of Penkenuma Swamp 5 were used instead of that of the Hitominuma Gj5 male, since their origin was thought to be the same (see text).
