# Supplementary material for: Possible Shifts in the Genetic Diversity of Red-crowned Cranes (Grus japonensis) in Hokkaido, Japan: Indications of Continental Gene Flow
Source: Animals (Basel). 2024 May 30;14(11):1633. doi: 10.3390/ani14111633 (PMC11171382; doi:10.3390/ani14111633)
Supplement: Supplementary file 1 [file animals-14-01633-s001.zip › animals-3026866-supplementary.pdf]

Table S1. Muscle and blood samples collected in southeastern Hokkaido used in this study.

	Time	Subprefecture	Municipalities	Longitude latitude	Stage	Tissue	Note
R566	2021-02-10	Kushiro	Onbetsu	-	Adult	Muscle	T74
R568	2031	Kushiro	Onbetsu	-	Adult	Muscle	T73
63	2007-07-29	Tokachi	Urahoro	42° 43’26"N 143° 38'12" E	Chick	Blood	
70	2008-06-22	Kushiro (13)	Kurhiro	43° 03'04.0"N 144° 13'10.0"E	Chick	Blood	
72	2008-06-29	Nemuro (13)	Nemuro	43° 16'50.0"N 145° 18'20.0"E	Chick	Blood	
109	2009-07-04	Tokachi (13)	Urahoro	42° 44'39.0"N 143° 35'59.0"E	Chick	Blood	
116	2009-07-12	Kushiro	Hamanaka	43° 11'59.0"N 145° 11'52.0"E	Chick	Blood	
123	2009-07-06	Kushiro (13)	Shiranuka	42° 59'58.0"N 144° 09'36.0"E	Chick	Blood	
124	2009-07-20	Tokachi	Toyokoro	42° 45'59.0"N 143° 34'23.0"E	Chick	Blood	
125	2009-07-20	Tokachi	Toyokoro	42° 45'59.0"N 143° 34'23.0"E	Chick	Blood	
131	2010-07-03	Tokachi (13)	Toyokoro	42° 51'30.0"N 143° 27'08.0"E	Chick	Blood	
137	2010-07-11	Nemuro (13)	Nemuro	43° 14'40.0"N 145° 31'40.0"E	Chick	Blood	
139	2010-07-24	Nemuro (13)	Nemuro	43° 16'50.0"N 145° 18'20.0"E	Chick	Blood	
169	2012-06-30	Tokachi	Taiki	42° 35'28.0"N 143° 28'37.0"E	Chick	Blood	
201	2013-07-15	Tokachi (13)	Urahoro	42° 43'03.0"N 143° 36'59.0"E	Chick	Blood	
204	2013-07-21	Kushiro	Hamanaka	43° 12'08.0"N 145° 11'27.0"E	Chick	Blood	
207	2014-06-28	Tokachi (13)	Ikeda	42° 58'36.0"N 143° 26'03.0"E	Chick	Blood	
213	2013-07-05	Tokachi (13)	Toyokoro	42° 44'45.0"N 143° 25'42.0"E	Chick	Blood	
221	2014-07-14	Kushiro	Shibecha	43° 06'43.0"N 144° 34'37.0"E	Chick	Blood	
223	2014-07-19	Nemuro (13)	Bekkai	43° 19'43.0"N 145° 20'05.0"E	Chick	Blood	
244	2015-06-30	Kushiro	Shiranuka	42° 57'53" N 144° 00'35" E	Chick	Blood	
245	2015-06-30	Kushiro	Shiranuka	42° 57'53" N 144° 00'35" E	Chick	Blood	
247	2015-07-01	Kushiro (13)	Hamanaka	43° 12'13" N 145° 00'43" E	Chick	Blood	
251	2015-07-03	Kushiro (13)	Shibecha	43° 16'56" N 144° 32'53" E	Chick	Blood	
253	2015-07-04	Kushiro	Shibecha	43° 11'17" N 144° 43'39" E	Chick	Blood	
254	2015-07-05	Tokachi (13)	Toyokoro	42° 45'41" N 143° 25'11" E	Chick	Blood	
256	2015-07-11	Tokachi (13)	Toyokoro	42° 51'25" N 143° 27'16" E	Chick	Blood	
261	2015-07-18	Kushiro	Hamanaka	43° 12'13" N 145° 11'26" E	Chick	Blood	
271	2016-07-02	Kushiro (13)	Shiranuka	43° 00'46" N 143° 58'22" E	Chick	Blood	
280	2016-07-10	Nemuro (13)	Bekkai	43° 19'50" N 145° 20'03" E	Chick	Blood	
287	2016-07-17	Kushiro	Hamanaka	43° 00'60" N 145° 00'29" E	Chick	Blood	
312	2016-07-08	Kushiro	Hamanaka	43° 12'16.0"N 145° 11'27.0"E	Chick	Blood	
313	2016-07-08	Kushiro	Hamanaka	43° 12'16.0"N 145° 11'27.0"E	Chick	Blood	

	Time	Subprefecture	Municipalities	Longitude latitude	Stage	Tissue	Note
315	2017-07-08	Kushiro (13)	Hamanaka	43° 10'12.0"N 145° 12'43.0"E	Chick	Blood	
316	2017-07-08	Kushiro	Hamanaka	43° 10'12.0"N 145° 12'43.0"E	Chick	Blood	
319	2017-07-16	Nemuro (13)	Bekkai	43° 26'58.0"N 145° 10'55.0"E	Chick	Blood	
330	2018-07-12	Nemuro (13)	Nemuro	43° 15'46.0"N 145° 29'14.0"E	Chick	Blood	
331	2018-07-08	Tokachi	Toyokoro	42° 52'04.0"N 143° 27'19.0"E	Chick	Blood	
344	2019-06-30	Kushiro (13)	Kushiro	43° 10'30.0"N 144° 12'59.0"E	Chick	Blood	
352	2019-07-06	Tokachi	Ikeda	43° 00'04.0"N 143° 26'58.0"E	Chick	Blood	
353	2019-07-07	Kushiro	Hamanaka	43° 12'16.0"N 145° 11'27.0"E	Chick	Blood	
358	2019-07-14	Kushiro	Hamanaka	43° 13'03.0"N 145° 10'58.0"E	Chick	Blood	
359	2019-07-14	Kushiro	Hamanaka	43° 13'03.0"N 145° 10'58.0"E	Chick	Blood	
361	2019-07-14	Kushiro (13)	Hamanaka	43° 12'10.0"N 145° 13'34.0"E	Chick	Blood	
362	2019-07-15	Kushiro (13)	Teshikaga	43° 32'48.0"N 144° 25'30.0"E	Chick	Blood	
371	2020-06-27	Tokachi (13)	Makubetsu	42° 55'10.2"N 143° 18'05.4"E	Chick	Blood	
373	2020-06-27	Tokachi (13)	Ikeda	42° 53'26.5"N 143° 26'33.4"E	Chick	Blood	
374	2020-06-28	Tokachi	Urahoro	42° 48'06.1"N 143° 36'29.5"E	Chick	Blood	
375	2020-06-28	Tokachi	Urahoro	42° 42'34.2"N 143° 38'17.5"E	Chick	Blood	
384	2020-07-04	Kushiro	Shibecha	43° 11'15.7"N 144° 43'40.1"E	Chick	Blood	
387	2020-07-07	Tokachi	Ikeda	42° 59'57.5"N 143° 27'04.3"E	Chick	Blood	
388	2020-07-07	Tokachi	Ikeda	42° 59'57.5"N 143° 27'04.3"E	Chick	Blood	
390	2020-07-09	Kushiro	Hamanaka	43° 12'19.1"N 145° 11'35.2"E	Chick	Blood	
392	2020-07-12	Tokachi	Honbetsu	43° 05'15.0"N 143° 30'26.3"E	Chick	Blood	
396	2020-07-15	Nemuro (13)	Bekkai	43° 25'21.0"N 145° 14'04.2"E	Chick	Blood	
397	2020-07-18	Nemuro (13)	Bekkai	43° 29'16.1"N 145° 00'44.6"E	Chick	Blood	
415	2021-07-01	Kushiro	Shibecha	43° 12'21.6"N 144° 42'39.6"E	Chick	Blood	
420	2021-07-03	Tokachi	Onbetsu	42° 54'16.2"N 143° 56'23.6"E	Chick	Blood	

Most blood samples were collected from chicks during the annual banding survey conducted in summer by the NPO Red-crowned Crane Conservancy. R566 (banding No. T74) and R568 (T73) are banded cranes that were found dead and are currently stored in the freezer of Kushiro City Zoo.

Table S2. InDel type of feathers and bloods collected in northern Hokkaido between 2010 and 2021.

Feather sample		Collection	Sex	Haplotype	Id-01	Id-02	Id-03	Id-04	Id-05	Id-06	Id-07	Id-08	Id-09	Id-10	Id-11
Hitominuma Swamp male (Gj5)	Father	Jul. 7, 2018	M	5	W-W	W-D	I-I	W-D	W-D	W-W	W-W	W-W	W-D	D-D	W-W
Penkenuma Swamp 5	Identical to the above	Sep. 8, 2021	M	5	W-W	W-D	I-I	W-D	W-D	W-W	W-W	W-W	W-D	D-D	W-W
Hitominuma Swamp female (Gj2)	Mother	Jul. 7, 2018	F	2	W-W	W-D	I-I	W-W	W-D	W-D	W-W	W-W	W-D	D-D	W-W
Penkenuma Swamp 13	Identical to the above	Sep. 8, 2021	F	2	W-W	W-D	I-I	W-W	W-D	W-D	W-W	W-W	W-D	D-D	W-W
Lake Kuccharo 1	Chick of Hitominuma Swamp pair	Jul. 2010	F	2	W-W	W-D	I-I	W-D	W-D	W-D	W-D	W-W	-	D-D	W-W
Lake Kuccharo 4	Chick of Hitominuma Swamp pair	Jul. 2010	F	2	W-W	-	I-I	W-D	W-W	W-D	W-D	W-W	W-D	D-D	W-W
Lake Kuccharo 6	Chick of Hitominuma Swamp pair	Jul. 2010	M	2	W-W	-	I-I	W-W	W-W	W-D	W-W	D-D	W-D	D-D	W-W
429 (Toyotomi)	Chick of Hitominuma Swamp pair	Nov. 22, 2021	M	2	W-W	W-W	I-I	W-W	W-W	W-W	W-W	W-W	W-W	W-D	W-W
Kabutonuma Swamp 3	Chick of Hitominuma Swamp pair	Sep. 27, 2021	F	2	W-W	W-D	I-I	W-W	W-D	W-W	W-W	W-W	W-W	-	W-W
Kabutonuma Swamp 5	Chick of Hitominuma Swamp pair	Sep. 27, 2021	M	2	W-W	W-D	I-I	W-W	-	W-W	W-W	W-W	W-W	D-D	W-W
Kabutonuma Swamp 6	Chick of Hitominuma Swamp pair	Sep. 27, 2021	F	2	W-W	W-D	I-I	W-D	W-D	W-W	W-W	W-W	W-D	-	W-W
Lake Kuccharo 2	Relative of Hitominuma Swamp pair	Jul. 2010	F	2	W-W	W-D	I-I	W-D	W-D	W-D	W-D	W-W	W-D	D-D	W-W
Lake Kuccharo 3	Relative of Hitominuma Swamp pair	Jul. 2010	M	2	W-W	W-D	I-I	W-D	W-D	W-D	W-D	W-W	W-D	D-D	W-W
Lake Kuccharo 5	Relative of Hitominuma Swamp pair	Jul. 2010	M	2	W-W	W-W	I-I	W-D	W-W	W-D	-	W-W	W-D	D-D	W-W
Lake Kuccharo 7	Relative of Hitominuma Swamp pair	Jul. 2010	F	2	W-W	-	I-I	W-D	W-W	W-D	-	W-W	W-D	D-D	W-W
Lake Kuccharo 8	Relative of Hitominuma Swamp pair	Jul. 2010	F	2	-	-	I-I	W-D	W-D	-	W-W	W-W	-	D-D	W-W
449 (Toyotomi)		Oct. 25, 2022	M	U	W-W	-	I-I	W-W	W-W	W-W	W-W	W-W	-	D-D	W-W
Kabutonuma Swamp 1		Sep. 27, 2021	F	2	W-W	W-D	I-I	W-W	W-D	W-W	W-W	W-W	W-D	D-D	W-W
Kabutonuma Swamp 2		Sep. 27, 2021	U	2	W-W	W-D	I-I	W-W	W-D	W-D	W-W	W-W	W-D	D-D	W-W
Kabutonuma Swamp 4		Sep. 27, 2021	F	2	W-W	W-W	I-I	W-W	W-D	W-W	W-W	W-W	W-W	D-D	W-W
Penkenuma Swamp 2		Sep. 8, 2021	U	2	W-W	-	I-I	W-W	W-D	W-D	W-D	W-W	W-W	W-D	W-W
Penkenuma Swamp 4		Sep. 8, 2021	U	2	W-W	-	I-I	W-W	W-W	W-W	W-D	W-W	W-D	D-D	W-W
Penkenuma Swamp 6		Sep. 8, 2021	U	2	W-W	-	I-I	W-D	W-D	W-D	-	-	W-D	D-D	W-W
Penkenuma Swamp 7		Sep. 8, 2021	U	2	W-W	-	I-I	W-W	W-D	W-D	-	-	W-D	D-D	W-W
Penkenuma Swamp 8		Sep. 8, 2021	U	2	W-W	-	W-I	W-W	-	W-D	-	-	-	D-D	W-W
Penkenuma Swamp 9		Sep. 8, 2021	U	2	W-W	W-W	I-I	W-W	W-D	W-D	D-D	W-W	W-D	D-D	W-W
Penkenuma Swamp 10		Sep. 8, 2021	U	2	W-W	-	I-I	W-D	W-D	W-D	W-W	W-W	-	W-D	W-W
Penkenuma Swamp 11		Sep. 8, 2021	U	2	W-W	-	I-I	W-D	W-D	W-D	-	-	W-W	W-D	W-W
Penkenuma Swamp 12		Sep. 8, 2021	U	2	W-W	-	W-I	W-D	W-D	W-W	D-D	W-W	W-D	D-D	W-W
Penkenuma Swamp 14		Sep. 8, 2021	U	2	W-W	-	W-I	W-D	W-D	-	-	W-W	W-D	D-D	W-W
Penkenuma Swamp 15		Sep. 8, 2021	F	2	W-W	W-D	W-I	W-W	W-D	W-W	W-W	W-W	-	D-D	W-W
Penkenuma Swamp 17		Sep. 8, 2021	U	2	-	-	W-I	W-D	W-D	-	-	-	W-D	D-D	W-W

W, I and D are abbreviations of wild allele, insertion allele and deletion allele, respectively. (-) indicates no data. W Homo, I Homo and D Homo indicate the homogenous type of wild, insertion and deletion, respectively. WI and WD mean heterogeneous type of wild-insertion and wild-deletion, respectively. White for wild type homo (W Homo), dark green for deletion type homo (D-D), light green for hetero of wild type and deletion type (W-D), dark blue for insertion type homo (I-I) and light blue for hetero of wild type and insertion type (W-I) are indicated. With the exception of blood from No. 429 and No. 449, all other feathers found on the ground were used for analysis. Because of many deficient information for some feather samples, the combinations of Penkenuma Swamp 10 and 11, Penkenuma 12, 14, and 17, Lake Kuccharo 1 and Penkenuma Swamp 6, Penkenuma Swamp 7 and Kabutonuma Swamp 2 were not con-firmed as separate individuals by InDel type, sex, or haplotype.

Table S3. Amino acid sequences of MHC class I exon 3 molecules from red-crowned cranes in Hokkaido, Japan.

Allele	Sequence (positions 1–140)
Grja-UA*27a	AQTRQSTYGCELLEDGSTRGYWQDAYDGRDFIAFDTDTMTFTAADAAAQITKRKWEADGTVAEKWKHYLQNTCVEWLRKYVSYGQTVLEKKGEGETGTPGTGQRGRVCQPSPPPPPQSAPRSECWGRRPPGS*PCTAALT
Grja-UA*28a1-5	·H·L·RMV··D··········S·················M······································E··R·················AM··R·····M··········M···················R·········*·······
Grja-UA*28b	·H·L·RMV··D··········S·················M······································E··R·················AM··R·····M····················L···············Q·········*·······
Grja-UA*33a1-3	·H·L·RMV··D············F···············M······································E··R·················A··KR·································N······R·········*·······
Grja-UA*38a1-2	·H·V·RM···D··········L·················M··········GA······························R·················A···R···············H··················T·····R·········*·······
Grja-UA*44a1-2	·Y·L·CMV··D··········S·F···············M······································E··RQ···············C·····A···R········GQR·RVCQPSP·P··QSAPRSECRGRRPPGS*PCTAALT·S·
Grja-UA*47a1-2	·H·L·RMV··E··········S·F···············M······································E··RQ·····················A···R········GQR·RVCQPSP·P··QSAPRSECRGRRPPGS*PCTAALT·S·
Grja-UA*47b1-2	·H·L·RMV··E··········S·F···············M······································E··RQ·····················A···R··································R·········*·······
Grja-UA*50a1-3	·H·W·CMV··D··········S·F···············M······································E··RQ·····················A·············GQR·RVCQPSP·P··QSAPRSECRGRRPPGS*PCTAALT·S·
Grja-UA*51a1-4	·····RMV··D····························M···M··································E··RQ·····················A··································R····L··*·······
Grja-UA*54a1-2	·····RMV··D····························M······································E··R···························KR··································R·········*·······
Grja-UA*55a1-2	·H·L·RM···D··········S·················M···R···L·························R·········*·······
Grja-UA*57	·····RMV·······························M···M··································E··RQ·····················A·············P·························R·········*·······
Grja-UA*58a1-2	·H·L·RMV··D··········S·F···············M······································E··R······················A···R········GQR·RVCQPSP·P··QSAPRSECRGRRPPGS*PCTAALT·S·
Grja-UA*59a1-2	·H·L·RMV··D····························M······································E··RQ·····················A···R·······························N······R·········*·······
Grja-UA*60a1-2	·H·L·RM···D····························M···RQ·····················A···R········L·························R·········*·······
Grja-UA*61a1-2	······M···D··········L·················M···R······················A···R········L·························R·········*·······
Grja-UA*62a1-2	·H·W·CMV·············S·F···············M···R······················A···R········GQR·RVCQPSP·P··QSAPRSECRGRRPPGS*PCTAALT·S·
Grja-UA*63a1-2	·····RM···D····························M···M··································E··RQ·····················A··································R····L··*·······
Grja-UA*64	·H·L·RM···D····························M···RQ·····················A··································R·········*·······
Grja-UA*65	·H·W·HMI··D·····G····L·I···············M··········GA··························E··R······················A···R·····················L···············Q·········*·······
Grja-UA*66	·····RMV·······························M···M··································E··RQ·····················A··································R·········*·······
Grja-UA*67	·H·L·RM················F···············M··········G···························E··RQ·····················A···R··································R·········*·······
Grja-UA*68	······M··············S·F···············M···R······················A···R········L·························R·········*·······
Grja-UA*69	·····RMV··D····························M··········GA······························R······················A··································R····L··*·······
Grja-UA*70	·H·L·RM···D····························M···R······················A···R········L·························R·········*·······
Grja-UA*71	·H·L·RMV··E··········S·F···············M······································E··RQ···············C·····A···R········GQR·RVCQPSP·P··QNAPRSECRGRRPPGS*PCTAALT·S·
Grja-UA*72	·R···RMI··D····N·····R·I···············V······································E·QR·····KD··IK··········A···R····AM·MW·VAGT·VGAV·HRH·L·RAPRGPSVGEGGLRDADLVLPR·R
Grja-UA*73	·Y·L·CMV··D··········S·F···············M···M··································E··RQ·····················A···R········L·························R·········*·······
Grja-UA*74	·H·L·RMV··D··········S·················M······································E··RQ·······································L·························R·········*·······
Grja-UA*75	·H·L·RMV··D··········S·F···············M······································E··RQ·····················A···R········GQR·RVCQPSP·P··QSAPRSECRGRRPPGS*PCTAALT·S·
Grja-UA*76	·H·W·HMI··D·····G······F···············M··········G···························E··RQ·····················A···R··································R·········*·······
Grja-UA*77	·H·L·RM···D··········S·F···············M······································E··RQ·····················A···R·······························N······R·········*·······
Grja-UA*78	·H·L·RM···D····························M·······P··································RQ·····················A···R········L·························R·········*·······
Grja-UA*79	·H·L·RMI··D····N·······KG··············M····················V·················E·QR·····KD··IK·········RA···R····A···W·VAGT·ADAV·HRH·L·RAPRGPSVGEGGLRDADLVLPR·R

Since MHC types (MHC class I Exon 3) in the previous reports (Grja-UA*27-*56: Xu et al., 2022)) are shown up to 91 amino acid sequences (AA) (shaded), MHC types with different AA after the 92nd are distinguished by a and b. If the deduced amino acid sequence was the same but the nucleotide sequence was different, it was designated as a1-a5, for example. GrjaUA*57 -*79 are novel MHC types identified in this study. The dots (midpoints) indicate the identity with the reference sequence at the top (Grja-UA*27). Red asterisks indicate amino acid deletions due to stop codons.

Table S4. Comparison of nucleotide type of MHC in a family of red-crowned crane in southeastern Hokkaido.

		Grja-UA*	28a1	28a3	47a1	50a1	51a1	54a1	54a2	55a1	55a2	57	58a1	58a2	59a1	59a2	64	66
R123	Male parent		blue		blue	blue	blue	blue		blue		blue	blue		blue		blue	blue
R131	Female parent							red	red	red	red		red	red	red	red		
		% of northern cranes	48.6	8.6	68.6	51.4	40.0	2.9	0.0	2.9	0.0	51.4	5.7	2.9	8.6	2.9	0.0	5.7
		% of southeastern cranes	41.4	10	72.4	53.4	32.8	39.7	17.2	39.7	17.2	53.4	41.4	29.3	37.9	13.8	20.7	15.5
		% of total cranes	44.1	9.7	71.0	52.7	35.5	25.8	10.8	25.8	10.8	52.7	28.0	19.4	26.9	9.7	12.9	11.8
244	Male chick		blue	yellow	blue	blue			red	purple		blue	purple		purple		blue	
245	Female chick							purple	red	purple	red		purple	red		blue		

A family of red-crowned crane consisting of parents and male and female chicks were found based on banding record. Nucleotide type of MHC (MHC class I Exon 3) were compared. Blue box indicates that the same MHC type was detected as in their father. Red box indicates the same MHC type as their mother. Purple boxes indicate the same MHC type as their parents, and yellow boxes indicate no MHC type matching their parents. "% of total cranes" represents the detection rate among 89 cranes examined in this study. "*28a1" in the top row means Grja-UA*28a1 for example.

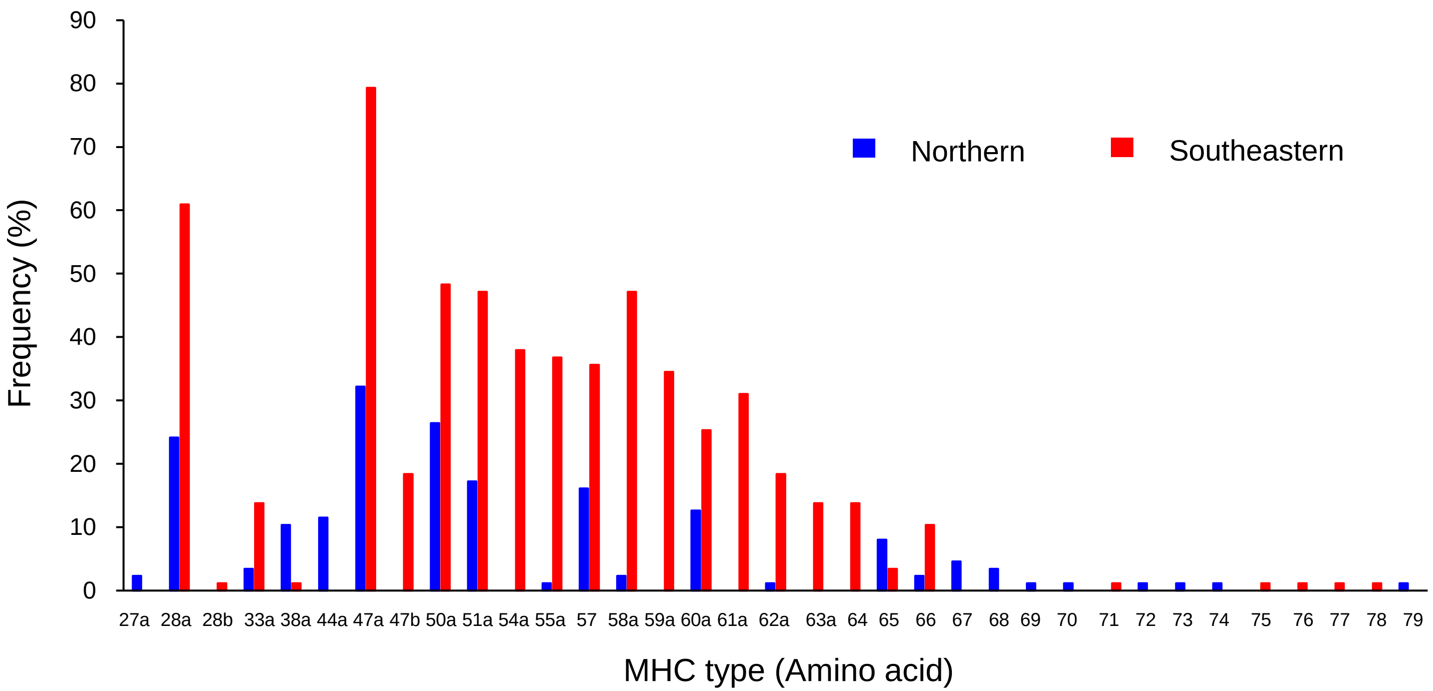

Figure S1. Frequencies of MHC class I Exon 3 types based on AA sequence in red-crowned crane in Hokkaido. Distributions and frequencies of each type in the northern (29 cranes, blue) and southeastern (58 cranes, red) population (total 89 cranes). Of the 32 feather samples from northern Hokkaido (Table S1), 29 were used to create a graph. The exceptions were the Hitominuma Swamp Gj5 male, whose MHC type was barely detected, and the Penkenuma Swamp 13, which appeared to originate from the Hitominuma Swamp Gj2 female. Therefore, as mentioned in Table S2, some samples may have come from the same individual. A total of 58 samples of all muscle and blood examined for MHC were included. “27” means Grja-UA*27 for example.
